# State Socioeconomic Indicators and Self-Reported Hypertension Among US Adults, 2011 Behavioral Risk Factor Surveillance System

**DOI:** 10.5888/pcd12.140353

**Published:** 2015-02-26

**Authors:** Amy Z. Fan, Sheryl M. Strasser, Xingyou Zhang, Jing Fang, Carol G. Crawford

**Affiliations:** Author Affiliations: Sheryl M. Strasser, School of Public Health, Georgia State University, Atlanta, Georgia; Xingyou Zhang, Jing Fang, Carol G. Crawford, Centers for Disease Control and Prevention, Atlanta, Georgia.

## Abstract

**Introduction:**

Hypertension is the leading cause of chronic disease and premature death in the United States. To date, most risk factors for hypertension have been identified at the individual (micro) level. The association of macro-level (area) socioeconomic factors and hypertension prevalence rates in the population has not been studied extensively.

**Methods:**

We used the 2011 Behavioral Risk Factor Surveillance System to examine whether state socioeconomic status (SES) indicators predict the prevalence of self-reported hypertension. Quintiles of state median household income, unemployment rate among the population aged 16 to 64 years, and the proportion of the population under the national poverty line were used as the proxy for state SES. Hypertension status was determined by the question “Have you ever been told by a doctor, nurse, or other health professional that you have high blood pressure?” Logistic regression was used to assess the relationship between state SES and hypertension with adjustment for individual covariates (demographic and socioeconomic factors and lifestyle behaviors).

**Results:**

States with a median household income of $43,225 or less (odds ratio [95% confidence interval] = 1.16 [1.08–1.25]) and states with 18.7% or more of residents living below the poverty line (odds ratio [95% confidence interval] = 1.14 [1.04–1.24]) had a higher prevalence of hypertension than states with the most residents in the most advantageous quintile of the indicators.

**Conclusion:**

The observed state SES–hypertension association indicates that area SES may contribute to the burden of hypertension in community-dwelling adults.

## MEDSCAPE CME

Medscape, LLC is pleased to provide online continuing medical education (CME) for this journal article, allowing clinicians the opportunity to earn CME credit.

This activity has been planned and implemented in accordance with the Essential Areas and policies of the Accreditation Council for Continuing Medical Education through the joint sponsorship of Medscape, LLC and Preventing Chronic Disease. Medscape, LLC is accredited by the ACCME to provide continuing medical education for physicians.

Medscape, LLC designates this Journal-based CME activity for a maximum of 1 ***AMA PRA Category 1 Credit(s)™***. Physicians should claim only the credit commensurate with the extent of their participation in the activity.

All other clinicians completing this activity will be issued a certificate of participation. To participate in this journal CME activity: (1) review the learning objectives and author disclosures; (2) study the education content; (3) take the post-test with a 75% minimum passing score and complete the evaluation at www.medscape.org/journal/pcd; (4) view/print certificate.

**Release date: February 26, 2015; Expiration date: February 26, 2016**

### Learning Objectives

Upon completion of this activity, participants will be able to:

- Assess the relationship between state median household income levels and the rate of hypertension
- Analyze the association between other socioeconomic variables and the prevalence of hypertension
- Distinguish population subgroups particularly affected by the relationship between socioeconomic variables and hypertension
- Identify the employment condition most affected by the relationship between socioeconomic variables and hypertension

**EDITORS**

Camille Martin, Editor, *Preventing Chronic Disease*. Disclosure: Camille Martin has disclosed no relevant financial relationships.

**CME AUTHOR**

Charles P. Vega, MD, Clinical Professor of Family Medicine, University of California, Irvine. Disclosure: Charles P. Vega, MD, has disclosed the following relevant financial relationships: Served as an advisor or consultant for: Lundbeck, Inc.; McNeil Pharmaceuticals; Takeda Pharmaceuticals North America, Inc.

**AUTHORS AND CREDENTIALS**

Disclosures: Amy Z. Fan, MD, PhD; Sheryl M. Strasser, PhD; Xingyou Zhang, PhD; Jing Fang, MD; Carol G. Crawford, PhD have disclosed no relevant financial relationships.

Affiliations: Sheryl M. Strasser, School of Public Health, Georgia State University, Atlanta, Georgia; Amy Z. Fan, Xingyou Zhang, Jing Fang, Carol G. Crawford, Centers for Disease Control and Prevention, Atlanta, Georgia.

## Introduction

Hypertension, also known as high blood pressure, is a significant health concern in the United States. According to the data from the biennial National Health and Nutrition Examination Survey from 2003 to 2010, 67 million adults (31%) reported having high blood pressure (1). In 2010, hypertension was listed as the primary cause of death of more than 360,000 Americans (2). The Framingham Heart Study showed that 90% of adults aged 55 and 65 years will develop hypertension within their lifetimes (3). Although hypertension is an independent health diagnosis, it is also a major risk factor for heart disease, stroke, congestive heart failure, and kidney disease (4).

Socioeconomic status (SES) can be assessed at both the micro (individual) level and the macro (community, state, or national) level. The literature supports clear and strong associations between micro-level SES measures (eg, income, poverty, education, type of employment, lack of insurance), with risk for, prevalence of, and ability to treat hypertension (5–8). A few studies have examined the association of macro-level SES with blood pressure or hypertension, and the findings are mixed (5,9,10). Most studies examined neighborhood contexts, which are smaller units than states. The objective of this study was to investigate whether state SES indicators are predictive of the prevalence of hypertension independent of the individual demographic, socioeconomic, and lifestyle risk factors.

## Methods

### Data sources and state variables

The Behavioral Risk Factor Surveillance System (BRFSS), launched by the Centers for Disease Control and Prevention (CDC) in 1984, is an ongoing, state-based surveillance system that conducts telephone health surveys of noninstitutionalized US adults aged 18 years or older in all 50 states, the District of Columbia, and US territories. The number of US households that have a cellular telephone but no landline telephone is rising steadily. To maintain survey coverage and validity, BRFSS has included cellular telephones in its samples since 2011 (11). In addition, beginning with the 2011 dataset, raking (iterative proportional fitting) succeeded poststratification as the sole BRFSS statistical weighting method to account for discrepancies between the demographic characteristics of respondents and the target population caused by declining response rates (12). Response rates for BRFSS are calculated according to standards set by the American Association of Public Opinion Research Response Rate formula number four (13). The response rate is the number of respondents who completed the survey as a proportion of all eligible and likely eligible persons. Data from US territories were not included in our analysis. The median landline survey response rate for all states and Washington, DC, in 2011 was 53.0% and ranged from 37.4% to 66.5%. The median cellular telephone survey response rate for all states and Washington, DC, in 2011 was 27.9% and ranged from 20.2% to 54.0%. The median of the combined weighted response rate for all states and Washington, DC, in 2011 was 49.7% and ranged from 33.8% to 64.1% (14). More information on data collection, quality control, and other survey or analytic methodologic procedures can be found on the BRFSS website (www.cdc.gov/brfss). We restricted our analysis to the data from respondents with no missing values for any covariate or the dependent variable (N = 446,137).

The American Community Survey (ACS) is a nationwide survey conducted in all US counties. The ACS collects and produces economic, social, demographic, and housing information annually. Approximately 3 million housing unit addresses are sampled annually throughout the United States and Puerto Rico. Data for 3 state SES variables were obtained from the 2011 ACS: 1) percentage of people below the national poverty level in the previous 12 months, 2) unemployment rate among the population aged 16 to 64 years, and 3) median household income. A higher proportion of people below the national poverty level, low employment-to-population ratio, and low median household income indicate economically distressed states. We obtained quintiles of these state variables and merged their quintile variables with BRFSS data by state.

All respondents were asked, “Have you ever been told by a doctor, nurse, or other health professional that you have high blood pressure?” Women who were hypertensive only during pregnancy were not regarded as having hypertension. All the other respondents who answered yes to the first question were categorized as having diagnosed hypertension.

### Individual covariates

**Consumption of fruits and vegetables per day.** Respondents were asked 6 questions to assess their consumption of fruits and vegetables: 1) “How often do you drink fruit juices such as orange, grapefruit, or tomato?” 2) “Not counting juice, how often do you eat fruits?” 3) “How often do you eat green salad?” 4) “How often do you eat potatoes, not including French fries, fried potatoes, or potato chips?” 5) “How often do you eat carrots?” 6) “Not counting carrots, potatoes, or salad, how many servings of vegetables do you usually eat? (Example: a serving of vegetables at both lunch and dinner would be 2 servings.)”

The response set included servings per day, week, month, or year; “never”; “don’t know/not sure”; and refusal. Servings of fruit or vegetable consumed were calculated separately. Total number of servings of fruits and vegetables per day was calculated. Quintiles of this variable were obtained.

**Body mass index (BMI) category.** Respondents’ BMI (weight [kg]/height [m^2^]) was calculated from their self-reported weight and height. Underweight is defined as a BMI of less than 18.5, normal weight is a BMI of 18.5 to 24.9, overweight is a BMI of 25.0 to 29.9, and obese is a BMI of 30.0 or higher.

**Smoking status.** Two questions were used to determine smoking status: “Have you smoked at least 100 cigarettes in your entire lifetime?” and “Do you now smoke cigarettes every day, some days, or not at all?” Respondents who reported having never smoked 100 cigarettes in their lifetime were categorized as never smokers. Respondents who reported having smoked 100 cigarettes in their lifetime and who were currently smoking were categorized as current smokers. Respondents who reported having smoked 100 cigarettes in their lifetime and who were not smoking now were categorized as former smokers.

**Exercise/leisure physical activity.** Respondents were asked “During the past month, other than your regular job, did you participate in any physical activities or exercises such as running, calisthenics, golf, gardening, or walking for exercise?” Those who answered no to this question were categorized as having engaged in no exercise/leisure physical activity.

**Heavy and binge drinking.** Four questions were used to assess whether a respondent had engaged in harmful alcohol consumption: 1) “During the past 30 days, have you had at least 1 drink of any alcohol beverages such as beer, wine, a malt beverage, or liquor?” 2) “During the past 30 days, how many days per week or per month did you have at least 1 drink of any alcohol beverage?” 3) “One drink is equivalent to 12 ounces of beer, a 5-oz glass of wine, or a drink with 1 shot of liquor. During the past 30 days on the days when you drank, about how many drinks did you drink on the average?” 4) “Considering all types of alcohol beverages, how many times during the past 30 days did you have x (x = 5 for men and 4 for women) or more drinks on an occasion?” Men who reported drinking more than 2 alcoholic beverages per day and women who reported drinking more than 1 alcoholic beverage per day were categorized as heavy drinkers. Men who reported having 5 or more drinks per occasion and women who reported having 4 or more drinks per occasion were categorized as binge drinkers.

**Other covariates.** Covariates included sex, age (18–34, 35–44, 45–54, 55–64, ≥65 years), race/ethnicity (non-Hispanic white, non-Hispanic black, other non-Hispanic, Hispanic), education attainment (less than high school graduate, high school graduate or equivalent, some college, college degree or more), marital status (married, previously married, never married), annual household income (<$15,000; $15,000–$24,999; $25,000–$34,999; $35,000–$49,999; ≥$50,000; do not know/missing), and employment status (employed, unemployed, retired, unable to work, other).

### Statistical analysis

The distributions of demographic variables in the study population were estimated by incorporating final combined landline telephone and cellular telephone weight in SAS-callable SUDAAN. The final weight in the BRFSS data was rescaled before hierarchical logistic regression models were fit with generalized linear mixed models (GLMM) using PROC GLIMMIX in SAS (SAS Institute, Inc). GLIMMIX procedure is a useful tool for hierarchical modeling with discrete responses. Two-level (individuals nested within states) random-intercept logistic models were used. Similar multilevel models have been used by other public health researchers (15,16). The associations between state socioeconomic factors and hypertension status were examined with adjustment for individual covariates. For comparison, we also fit logistic regression models using RLOGIST in SUDAAN (RTI International). Statistical significance was assessed by a Wald test at *P* < .05.

To assess the interaction between state SES variables and individual characteristics, we also constructed a series of GLMM with the following terms entered to predict the prevalence of hypertension: 1) individual characteristics (age, sex, race/ethnicity, employment status, education attainment, marital status, household income, fruit and vegetable intake, leisure physical activity, BMI category, smoking status, binge drinking habit, and heavy drinking habit), 2) the dummy state SES variable (dichotomized by regrouping the quintiles based on preceding analysis to facilitate interpretation of the results), and 3) the interaction term between an individual characteristic and the dummy state SES variable. These GLMM models were used to examine whether the effect of the state SES variables on our outcome depends on values of an individual characteristic. A *P* value of less than .05 indicates that the effect of a state SES variable differs significantly across categories of an individual characteristic. Analyses were performed using SAS version 13 or SAS-Callable SUDAAN version 13.2 (SAS Institute Inc).

## Results

The prevalence of self-reported hypertension was 32.8% in the United States in 2011 (standard error [SE], 0.1%). The prevalence varied by state, ranging from 24.1% (SE, 0.5%) in Utah to 40.9% (SE, 0.8%) in Alaska. 

The adjusted odds ratios (AORs; 95% confidence interval [CI]) from PROC GLIMMIX (Table 1) show that the following individual characteristics were associated with higher odds of self-reported hypertension: being male; being older; being non-Hispanic black or non-Hispanic other (rather than non-Hispanic white); being previously married; having low education attainment; having low annual household income; being unemployed, retired, or unable to work; having low consumption of fruits and vegetables; being overweight or obese; being a former or current smoker; having no leisure physical activity or exercise; and heavy or binge drinking.

**Table 1:** Association Between Demographic and Behavioral Characteristics and Self-Reported Hypertension Status, 2011 Behavioral Risk Factor Surveillance System (N = 446,137)^a^

Characteristic	% (SE)	Hypertension Prevalence, % (SE)	AOR (95% CI)
**Sex**			
Male	49.8 (0.2)	33.8 (0.2)	1.26 (1.24–1.27)
Female	50.2 (0.2)	31.3 (0.2)	1 [Reference]
**Age, y**			
18–34	29.8 (0.2)	10.8 (0.2)	0.09 (0.08–0.09)
35–44	17.5 (0.1)	21.3 (0.3)	0.17 (0.16–0.17)
45–54	19.2 (0.1)	34.5 (0.3)	0.30 (0.29–0.31)
55–64	15.6 (0.1)	50.2 (0.3)	0.56 (0.54–0.57)
≥65	17.8 (0.1)	63.5 (0.2)	1 [Reference]
**Race/ethnicity**			
White, non-Hispanic	67.8 (0.2)	33.5 (0.1)	1 [Reference]
Black, non-Hispanic	11.0 (0.1)	40.6 (0.5)	1.58 (1.54–1.62)
Hispanic	13.3 (0.1)	23.7 (0.4)	0.86 (0.83–0.88)
Other, non-Hispanic	7.9 (0.1)	28.3 (0.5)	1.15 (1.12–1.18)
**Marital status^b^**			
Married	50.7 (0.2)	34.2 (0.2)	1.05 (1.02–1.07)
Previously married	19.9 (0.1)	48.6 (0.3)	1.23 (1.20–1.26)
Never married	29.4 (0.2)	18.8 (0.3)	1 [Reference]
**Education**			
<High school graduate	14.3 (0.1)	37.3 (0.4)	1.23 (1.20–1.27)
High school graduate	29.1 (0.1)	35.8 (0.2)	1.18 (1.15–1.20)
Some college	30.5 (0.2)	31.6 (0.2)	1.17 (1.14–1.19)
≥College degree	26.1 (0.1)	26.8 (0.2)	1 [Reference]
**Annual household income, $**			
<15,000	11.1 (0.1)	32.4 (0.3)	1.17 (1.13–1.21)
15,000–24,999	16.0 (0.1)	37.4 (0.4)	1.12 (1.09–1.15)
25,000–34,999	10.1 (0.1)	36.8 (0.4)	1.10 (1.07–1.13)
35,000–49,999	12.4 (0.1)	36.0 (0.4)	1.07 (1.04–1.09)
≥50,000	38.4 (0.2)	33.7 (0.4)	1 [Reference]
Don’t know/not sure/missing	12.1 (0.1)	27.7 (0.2)	1.00 (0.98–1.03)
**Employment status**			
Employed	55.4 (0.2)	18.4 (0.3)	1 [Reference]
Unemployed	8.9 (0.1)	28.4 (0.5)	1.19 (1.16–1.23)
Retired	16.6 (0.1)	61.7 (0.2)	1.35 (1.32–1.39)
Unable to work	6.5 (0.1)	58.1 (0.6)	2.21 (2.14–2.28)
Other	12.6 (0.1)	24.8 (0.2)	0.94 (0.92–0.97)
**Quintiles of servings of fruit and vegetable intake per day**			
0−1.66	21.2 (0.1)	34.4 (0.3)	1.08 (1.06–1.11)
1.67−2.53	20.8 (0.1)	32.9 (0.3)	1.05 (1.03–1.08)
2.54−3.41	19.5 (0.1)	33.4 (0.3)	1.06 (1.04–1.09)
3.42−4.70	18.8 (0.1)	32.6 (0.3)	1.04 (1.01–1.06)
4.71−39.0	19.7 (0.1)	29.1 (0.3)	1 [Reference]
**Body mass index category (kg/m^2^)**			
Underweight (<18.5)	1.9 (0.1)	17.3 (0.8)	0.81 (0.75–0.86)
Normal weight (18.5–24.9)	35.8 (0.2)	20.1 (0.2)	1 [Reference]
Overweight (25.0–29.9)	27.6 (0.1)	33.6 (0.2)	1.83 (1.80–1.86)
Obese (≥30.0)	34.8 (0.2)	48.8 (0.3)	3.78 (3.71–3.86)
**Smoking status^c^**			
Current smoker	20.3 (0.1)	30.5 (0.3)	1.08 (1.06–1.10)
Former smoker	25.1 (0.1)	43.6 (0.3)	1.11 (1.10–1.14)
Never smoker	54.6 (0.2)	28.2 (0.2)	1 [Reference]
**Exercise**			
Yes	74.8 (0.1)	29.7 (0.2)	0.88 (0.87–0.90)
No	25.2 (0.1)	40.5 (0.3)	1 [Reference]
**Binge drinking^d^**			
Yes	18.1 (0.1)	23.9 (0.3)	1.06 (1.04–1.08)
No	81.9 (0.1)	34.2 (0.1)	1 [Reference]
**Heavy drinking^d^**			
Yes	6.5 (0.1)	29.8 (0.5)	1.26 (1.22–1.30)
No	93.5 (0.1)	32.7 (0.1)	1 [Reference]

Abbreviations: AOR, adjusted odds ratio; CI, confidence interval; SE, standard error.

a The estimates were obtained with all individual variables and state variables entered simultaneously in the model.

b Marital status: married includes people who are married or living with a partner; previously married includes people who are divorced, separated, or widowed.

c Never smokers are those who reported having never smoked 100 cigarettes in their lifetime; current smokers are those who reported having smoked 100 cigarettes in their lifetime and who currently smoke; former smokers are those who reported having smoked 100 cigarettes in their lifetime but who do not smoke now.

d Men who reported drinking more than 2 alcoholic beverages per day and women who reported drinking more than 1 alcoholic beverage per day were categorized as heavy drinkers. Men who reported having 5 or more drinks per occasion and women who reported having 4 or more drinks per occasion were categorized as binge drinkers.

According to the 2011 ACS, the proportion of people below the national poverty level in the previous 12 months ranged from 8.8% (New Hampshire) to 22.6% (Mississippi); the unemployment rate among the population 16 to 64 years of age ranged from 2.7% (North Dakota) to 12.1% (Michigan); and the median household income ranged from $36,919 (Mississippi) to $70,004 (Maryland). The association of these state socioeconomic indicators with the prevalence of self-reported hypertension was examined by GLIMMIX (Table 2). After adjustment for individual characteristics (age, sex, race/ethnicity, marital status, education attainment, annual household income, employment status, leisure physical activity or exercise, fruit and vegetable intake, BMI category, smoking status, binge drinking, and heavy drinking), adults who resided in states with a median household income ($43,225 or less) were associated with a 16% higher odds of hypertension compared with adults from states with median household income of $58,814 or more (AOR [95% CI] = 1.16 [1.08–1.25]). Adults who resided in states with 18.7% or more of the population living below the poverty line had 14% higher odds of hypertension compared with adults who resided in states having 11.8% or less of the population living below the poverty line (AOR [95% CI] = 1.14 [1.04–1.24]). State unemployment rate was not associated with the odds of prevalence of hypertension in multivariate-adjusted models. This variable was not included in further interaction effects analysis. The results from RLOGIST were consistent with those obtained from GLIMMIX.

**Table 2:** Estimates^a^ of Self-Reported Hypertension in Association with State Socioeconomic Indicators Among US Community-Dwelling Adults, 2011 Behavioral Risk Factor Surveillance System (N = 446,137)

Indicator	AOR (95% Confidence Interval), GLIMMIX^b^	AOR (95% Confidence Interval), RLOGIST^c^
**Median household income, $**		
≤43,225	1.16 (1.08–1.25)	1.22 (1.17–1.28)
43,226−46,438	1.00 (0.92–1.07)	1.04 (1.00–1.08)
46,439−51,704	0.93 (0.86–1.00)	0.99 (0.94–1.03)
51,705−58,813	0.94 (0.87–1.01)	0.99 (0.95–1.04)
≥58,814	1 [Reference]	1 [Reference]
**Population below national poverty line, %**		
≥18.7	1.14 (1.04–1.24)	1.14 (1.09–1.19)
16.5−18.6	1.09 (1.00–1.19)	1.08 (1.03–1.12)
13.9−16.4	1.03 (0.95–1.13)	1.03 (0.99–1.08)
11.9−13.8	0.95 (0.87–1.04)	0.92 (0.88–0.96)
≤11.8	1 [Reference]	1 [Reference]
**Unemployment rate, %**		
≥10.8	1.10 (0.99–1.22)	1.03 (0.99–1.07)
9.6−10.7	1.09 (0.99–1.21)	1.03 (0.99–1.07)
8.4−9.5	1.09 (0.98–1.20)	1.00 (0.96–1.04)
7.0−8.3	1.01 (0.92–1.12)	1.00 (0.95–1.04)
≤6.9	1 [Reference]	1 [Reference]

Abbreviation: AOR, adjusted odds ratio.

a The estimates were obtained with adjustment for individual characteristics, including age, sex, race/ethnicity, employment status, education attainment, marital status, household income, fruit and vegetable intake, leisure physical activity, body mass index category, smoking status, binge drinking, and heavy drinking. The state socioeconomic indicators were obtained from the 2011 American Community Survey.

b Generalized linear mixed models (GLMM) using PROC GLIMMIX in SAS (SAS Institute, Inc).

c SUDAAN’s RLOGIST procedure (RTI International).

We dichotomized the state poverty level (percentage of population below the national poverty line ≥16.5% vs others) and median household income (median household income ≤$43,225 vs others) by regrouping state quintiles based on the results presented above and conducted an interaction effects analysis (Table 3). Some significant interactions between individual and state variables were shown. For example, the low state SES appeared to impose stronger adverse effects on self-reported hypertension for women than for men. Adults aged 18–34 years and adults who never married did not seem to be significantly affected by state SES in terms of hypertension prevalence. Adults of Hispanic origin and adults with less than a high school education were not significantly affected by state poverty level. Adults who were unable to work were the most affected by low state SES of all employment status categories (30% and 38% higher odds of reporting hypertension for state-level high poverty and low household income, respectively).

**Table 3:** Association^a^ Between State Socioeconomic Status Variables and Individual Variables on Odds of Self-Reported Hypertension, 2011 Behavioral Risk Factor Surveillance System (N = 446,137)

Individual Characteristic	Population Below National Poverty Line ≥16.5% vs Others, AOR (95% CI)	*P* Value^b^	Median Household Income ≤$43,225 vs Others, AOR (95% CI)	*P* Value^b^
**Sex**				
Male	1.08 (1.02–1.15)	<.001	1.15 (1.08–1.22)	<.001
Female	1.16 (1.10–1.23)		1.27 (1.19–1.35)	
**Age, y**				
18–34	1.00 (0.94–1.07)	<.001	1.04 (0.97–1.12)	<.001
35–44	1.20 (1.12–1.28)		1.39 (1.29–1.49)	
45–54	1.15 (1.08–1.23)		1.25 (1.17–1.34)	
55–64	1.15 (1.08–1.22)		1.23 (1.15–1.32)	
≥65	1.11 (1.04–1.18)		1.16 (1.08–1.24)	
**Race/ethnicity**				
White, non-Hispanic	1.13 (1.07–1.20)	.003	1.22 (1.14–1.29)	.67
Black, non-Hispanic	1.08 (1.01–1.16)		1.18 (1.10–1.28)	
Hispanic	1.04 (0.96–1.12)		1.19 (1.08–1.32)	
Other, non-Hispanic	1.18 (1.09–1.28)		1.17 (1.07–1.29)	
**Marital status^c^**				
Married	1.14 (1.08–1.21)	<.001	1.22 (1.15–1.30)	.01
Previously married	1.15 (1.08–1.22)		1.22 (1.14–1.31)	
Never married	1.03 (0.97–1.10)		1.14 (1.06–1.23)	
**Education**				
<High school graduate	1.04 (0.97–1.11)	<.001	1.29 (1.20–1.39)	.003
High school graduate	1.16 (1.09–1.23)		1.22 (1.15–1.31)	
Some college	1.11 (1.05–1.18)		1.17 (1.09–1.25)	
≥College degree	1.13 (1.06–1.20)		1.17 (1.09–1.26)	
**Annual household income, $**				
<15,000	1.14 (1.06–1.22)	.003	1.24 (1.15–1.33)	.30
15,000–24,999	1.14 (1.07–1.22)		1.25 (1.16–1.35)	
25,000–34,999	1.08 (1.00–1.16)		1.18 (1.10–1.27)	
35,000–49,999	1.10 (1.03–1.18)		1.15 (1.07–1.25)	
≥50,000	1.15 (1.09–1.22)		1.20 (1.11–1.29)	
Don’t know/not sure/missing	1.05 (0.98–1.13)		1.21 (1.14–1.30)	
**Employment status**				
Employed	1.11 (1.05–1.18)	<.001	1.24 (1.14–1.35)	<.001
Unemployed	1.06 (0.98–1.14)		1.09 (1.00–1.18)	
Retired	1.11 (1.04–1.18)		1.16 (1.09–1.25)	
Unable to work	1.30 (1.20–1.40)		1.38 (1.27–1.50)	
Other	1.11 (1.03–1.20)		1.21 (1.13–1.28)	
**Quintiles of servings of fruit and vegetable intake per day**				
0−1.66	1.14 (1.07–1.22)	.53	1.21 (1.13–1.29)	.99
1.67−2.53	1.11 (1.04–1.18)		1.21 (1.13–1.30)	
2.54−3.41	1.12 (1.05–1.20)		1.20 (1.12–1.29)	
3.42−4.70	1.10 (1.03–1.18)		1.20 (1.12–1.29)	
4.71−39.0	1.13 (1.06–1.21)		1.21 (1.12–1.31)	
**Body mass index category (kg/m^2^)**				
Underweight (<18.5)	1.29 (1.12–1.49)	.15	1.24 (1.05–1.46)	.60
Normal weight (18.5–24.9)	1.13 (1.06–1.19)		1.20 (1.12–1.28)	
Overweight (25.0–29.9)	1.12 (1.05–1.19)		1.23 (1.15–1.31)	
Obese (≥30.0)	1.11 (1.05–1.18)		1.19 (1.12–1.28)	
**Smoking status^d^**				
Current smoker	1.11 (1.04–1.18)	.59	1.17 (1.10–1.26)	.25
Former smoker	1.13 (1.07–1.21)		1.21 (1.13–1.29)	
Never smoker	1.12 (1.05–1.19)		1.22 (1.15–1.30)	
**Exercise**				
Yes	1.11 (1.05–1.17)	.02	1.20 (1.13–1.28)	.80
No	1.15 (1.08–1.23)		1.21 (1.13–1.29)	
**Binge drinking^e^**				
Yes	1.06 (1.00–1.14)	.003	1.14 (1.05–1.23)	.01
No	1.13 (1.07–1.20)		1.22 (1.15–1.29)	
**Heavy drinking^e^**				
Yes	1.10 (1.01–1.19)	.51	1.12 (1.02–1.24)	.07
No	1.12 (1.06–1.19)		1.21 (1.14–1.29)	

Abbreviations: AOR, adjusted odds ratio; CI, confidence interval.

a The estimates were obtained from generalized linear mixed models. The following terms were entered as predictors of self-reported hypertension: 1) individual characteristics (age, sex, race/ethnicity, employment status, education attainment, marital status, household income, fruit and vegetable intake, physical inactivity, body mass index category, smoking status, binge drinking, and heavy drinking); 2) the dummy state SES variable; 3) the interaction term of an individual characteristic times a state SES variable.

b A *P* value of <.05 indicates that the effect of a state SES variable is significantly different across categories of an individual characteristic.

c Marital status: married includes people who are married or living with a partner; previously married includes people who have been divorced, separated, or widowed.

d Never smokers are those who reported having never smoked 100 cigarettes in their lifetime; current smokers are those who reported having smoked 100 cigarettes in their lifetime and who currently smoke; former smokers are those who reported having smoked 100 cigarettes in their lifetime but who do not smoke now.

e Men who reported drinking more than 2 alcoholic beverages per day and women who reported drinking more than 1 alcoholic beverage per day were categorized as heavy drinkers. Men who reported having 5 or more drinks per occasion and women who reported having 4 or more drinks per occasion were categorized as binge drinkers.

## Discussion

This study indicated that states with low median household incomes and high percentages of the population below the poverty line were significantly associated with high prevalence of self-reported hypertension independent of individual SES and other characteristics. These findings align with those of other studies that documented variations in cardiovascular morbidity and mortality risk across communities with differential socioenvironmental characteristics. For example, the Atherosclerosis Risk in Communities Study indicated that increased neighborhood disadvantage (low median household income, median house value, and occupational categories) was associated with increased systolic blood pressure (17).

There are large state variations in the prevalence of self-reported hypertension (ranging from 24.1% to 40.9% in 2011). Our early report showed that self-reported hypertension prevalence was higher in the southern United States than in other regions (18). Median household income is generally lower in the southern states than elsewhere in the United States (www.census.gov/hhes/www/income/data/statemedian/).

The mechanisms underlying the association of state SES indicators with hypertension prevalence remain to be explored. State SES may influence an individual’s health through the association with community and individual SES. In our study, the state SES characteristics were significantly associated with individual SES variables (data not shown). However, state SES may affect one’s health by shaping the quality and quantity of social services as well as the physical environment. The data showed that the state median household income level had adverse effects on self-reported hypertension superimposed over the individual household income (the effects are additive). The association of state SES with self-reported hypertension were consistent across all categories of fruit and vegetable intake, exercise, BMI, smoking, and heavy drinking status (ie, no interactions were found).

The literature documents interactions between macro-level and individual SES on health and health-related outcomes. For example, Wilson et al demonstrated the buffering effect of family SES on the negative health consequences of living in low-SES neighborhoods for healthy black adolescents (9). Evidence indicates that education and income do not translate into the same level of financial and housing opportunities for different ethnic groups (19,20). A middle-class person who lives in a poor community may remain exposed to suboptimal conditions associated with that community. In our study, we also found interactions between state and individual SES and demographic variables. Women (vs men) and adults who were unable to work (vs adults with other employment status) were more vulnerable to the effects of disadvantageous state SES on hypertension risk. Nonetheless, young adults and adults who had never married were less likely to be affected by disadvantageous state SES. This is understandable because these groups overall are resilient to hypertension risk, possibly because of young age. The accumulated effects of disadvantaged macro-level SES may not manifest until older age. Adults of Hispanic origin and adults with less than high school education were least affected by state poverty level. Adults of Hispanic origin may be more resilient to hypertension risk. This racial/ethnic group was 14% less likely to report hypertension than non-Hispanic whites (Table 1). An alternative explanation for the “Hispanic paradox,” however, may be lower awareness of hypertension because of limited access to the health care system (21). In addition, adults with less than a high school education did not seem to benefit from high state SES. Policy and program implications should be addressed if these interactions between state and individual attributes are verified in further studies.

Most research using BRFSS data did not account for the potential importance of state attributes in influencing individual outcomes. The state SES is not equivalent to individual data aggregated to the state level. The SES indicators from different levels may come from different sources and have their unique associations with and contribution to health outcomes. In this study, the individual and state SES variables were found to be significantly independently associated with the prevalence of hypertension. 

Our study has limitations. The status of diagnosed hypertension — as well as demographic and lifestyle behaviors — were all self-reported and are subject to recall bias and inaccuracies. We considered only a limited battery of state SES variables and did not consider other contextual variables including environmental factors such as public park areas, crime rate (which can affect people’s access to and engagement in outdoor activities), access to healthful foods, and the local density of fast-food restaurants. These factors may influence the prevalence of hypertension in the population. For convenience, we sometimes illustrated the findings assuming causal inference. However, we could not draw conclusions on causal relationships between the predictors/interactions and the outcome because of the cross-sectional nature of BRFSS. The results from the state data may not directly be used to design community-level interventions. In addition, the findings of this study may not be generalizable to geographic areas other than the United States.

Prevention and control of hypertension are key elements in CDC’s state program for chronic disease prevention (22). Identification of risk factors of hypertension, at the individual and macro level, could guide efforts to optimize public health interventions. Together with other reports underscoring the importance of neighborhood characteristics, this study further suggests that hypertension risk may be influenced by societal structures, institutions, norms, and policies that may underlie geographic differences in chronic disease risk. Further comprehensive studies should examine how communities with similar or different demographic or socioeconomic profiles may have distinct disease patterns and whether those differences can be explained by differences in health care access, equitable policies, social norms, comprehensive public health capacities, and scope of services. Examination of interactions between determinants at different levels can also be used to design targeted interventions. Lifestyle modification programs for individuals may achieve better results if evidence-based community-level intervention components are incorporated.

## Figures and Tables

**Table:** 

**1. The activity supported the learning objectives.**				
**Strongly Disagree**				**Strongly Agree**
1	2	3	4	5
**2. The material was organized clearly for learning to occur.**				
**Strongly Disagree**				**Strongly Agree**
1	2	3	4	5
**3. The content learned from this activity will impact my practice.**				
**Strongly Disagree**				**Strongly Agree**
1	2	3	4	5
**4. The activity was presented objectively and free of commercial bias.**				
**Strongly Disagree**				**Strongly Agree**
1	2	3	4	5

## Post-Test Information

To obtain credit, you should first read the journal article. After reading the article, you should be able to answer the following, related, multiple-choice questions. To complete the questions (with a minimum 75% passing score) and earn continuing medical education (CME) credit, please go to http://www.medscape.org/journal/pcd. Credit cannot be obtained for tests completed on paper, although you may use the worksheet below to keep a record of your answers. You must be a registered user on Medscape.org. If you are not registered on Medscape.org, please click on the "Register" link on the right hand side of the website to register. Only one answer is correct for each question. Once you successfully answer all post-test questions you will be able to view and/or print your certificate. For questions regarding the content of this activity, contact the accredited provider, CME@medscape.net. For technical assistance, contact CME@webmd.net. American Medical Association's Physician's Recognition Award (AMA PRA) credits are accepted in the US as evidence of participation in CME activities. For further information on this award, please refer to http://www.ama-assn.org/ama/pub/about-ama/awards/ama-physicians-recognition-award.page. The AMA has determined that physicians not licensed in the US who participate in this CME activity are eligible for ***AMA PRA Category 1 Credits*** ™. Through agreements that the AMA has made with agencies in some countries, AMA PRA credit may be acceptable as evidence of participation in CME activities. If you are not licensed in the US, please complete the questions online, print the AMA PRA CME credit certificate and present it to your national medical association for review.

## Post-Test Questions

### Article Title: State Socioeconomic Indicators and Self-Reported Hypertension Among US Adults, 2011 Behavioral Risk Factor Surveillance System

**CME Questions**

1. What was the relationship between household income and the rate of hypertension in the current study by Fan and colleagues?
  - There was no significant relationship between income and hypertension
  - Low income was associated with higher rates of hypertension
  - Both middle and high incomes were associated with higher rates of hypertension
  - Only high incomes were associated with higher rates of hypertension
2. What was the relationship between other socioeconomic variables and the rate of hypertension in the current study by Fan and colleagues?
  - The poverty rate was unrelated to the prevalence of hypertension
  - Hypertension was more common in states with a higher rate of persons living in poverty
  - Higher rates of unemployment were associated with higher rates of hypertension
  - Any state with an unemployment rate above 7% had a higher rate of hypertension
3. Socioeconomic variables were particularly relevant in their association with hypertension in which of the following groups?
  - Women
  - Young adults
  - Adults who were never married
  - Hispanics
4. Lower socioeconomic status was ***most*** significantly associated with a higher rate of hypertension among which of the following employment categories?
  - Currently employed
  - Currently unemployed
  - Retired
  - Unable to work

**Evaluation**
